# Maternal Preconception Body Mass Index Overtakes Age as a Risk Factor for Gestational Diabetes Mellitus

**DOI:** 10.3390/jcm12082830

**Published:** 2023-04-12

**Authors:** Maria Mirabelli, Vera Tocci, Alessandra Donnici, Stefania Giuliano, Paola Sarnelli, Alessandro Salatino, Marta Greco, Luigi Puccio, Eusebio Chiefari, Daniela Patrizia Foti, Antonio Brunetti

**Affiliations:** 1 Department of Health Sciences, University “Magna Græcia” of Catanzaro, 88100 Catanzaro, Italy; maria.mirabelli@unicz.it (M.M.); tocci.vera@gmail.com (V.T.); salatino@unicz.it (A.S.); echiefari@unicz.it (E.C.); 2 Operative Unit of Endocrinology, “Mater Domini” University Hospital, 88100 Catanzaro, Italy; alessandradonnici@libero.it (A.D.); stefania.giuliano75@gmail.com (S.G.); 3 Operative Unit of Endocrinology, “Pugliese Ciaccio” Hospital, 88100 Catanzaro, Italy; paola.sarnelli.ps@gmail.com (P.S.); puccio55@libero.it (L.P.); 4 Department of Experimental and Clinical Medicine, University “Magna Græcia” of Catanzaro, 88100 Catanzaro, Italy; foti@unicz.it

**Keywords:** body mass index, obesity, advanced maternal age, gestational diabetes risk, insulin resistance

## Abstract

Introduction—The purpose of this study was to determine the relative impact of modifiable and non-modifiable risk factors in the development of gestational diabetes mellitus (GDM), with a particular focus on maternal preconception body mass index (BMI) and age, two important determinants of insulin resistance. Understanding the factors that contribute most to the current escalation of GDM rates in pregnant women could help to inform prevention and intervention strategies, particularly in areas where this female endocrine disorder has an elevated prevalence. Methods—A retrospective, contemporary, large population of singleton pregnant women from southern Italy who underwent 75 g OGTT for GDM screening was enrolled at the Endocrinology Unit, “Pugliese Ciaccio” Hospital, Catanzaro. Relevant clinical data were collected, and the characteristics of women diagnosed with GDM or with normal glucose tolerance were compared. The effect estimates of maternal preconception BMI and age as risk factors for GDM development were calculated through correlation and logistic regression analysis by adjusting for potential confounders. Results—Out of the 3856 women enrolled, 885 (23.0%) were diagnosed with GDM as per IADPSG criteria. Advanced maternal age (≥35 years), gravidity, reproductive history of spontaneous abortion(s), previous GDM, and thyroid and thrombophilic diseases, all emerged as non-modifiable risk factors of GDM, whereas preconception overweight or obesity was the sole potentially modifiable risk factor among those investigated. Maternal preconception BMI, but not age, had a moderate positive association with fasting glucose levels at the time of 75 g OGTT (Pearson coefficient: 0.245, *p* < 0.001). Abnormalities in fasting glucose drove the majority (60%) of the GDM diagnoses in this study. Maternal preconception obesity almost tripled the risk of developing GDM, but even being overweight resulted in a more pronounced increased risk of developing GDM than advanced maternal age (adjusted OR for preconception overweight: 1.63, 95% CI 1.320–2.019; adjusted OR for advanced maternal age: 1.45, 95% CI 1.184–1.776). Conclusions—Excess body weight prior to conception leads to more detrimental metabolic effects than advanced maternal age in pregnant women with GDM. Thus, in areas in which GDM is particularly common, such as southern Italy, measures aiming to counteracting maternal preconception overweight and obesity may be efficient in reducing GDM prevalence.

## 1. Introduction

Gestational diabetes mellitus (GDM) is a common female endocrine disorder that can occur during pregnancy. It is associated with an increased risk of perinatal mortality and morbidity, making it a major public health concern [1]. The prevalence of GDM has increased in recent decades, most probably due to factors such as older maternal age at conception and adherence to unhealthy dietary habits and lifestyles that predispose to excess body weight [2,3]. It is estimated that globally, one in every six pregnant women develops GDM [4]. However, the prevalence of GDM varies greatly among different ethnic groups and different screening methods and glucose thresholds used for diagnosing the condition. Hispanic, African American, Native American, South Asian, and Pacific Islander women have the highest prevalence of GDM, similar to women living in southern Italy [2]. Since 2010, the adoption of 75 g OGTT glucose thresholds from the International Association of Diabetes and Pregnancy Study Groups (IADPSG) consensus has resulted in a GDM diagnosis rate of up to 28% in this region [5]. Pregnant women with GDM are at greater risk of developing cardiovascular disease and type 2 diabetes mellitus (T2DM) over their lifetime. Furthermore, their newborns are more likely to suffer from obesity, T2DM, and other metabolic abnormalities in childhood, adolescence, and adulthood [1,2]. It is therefore urgently necessary to take preventive measures to reduce the number of cases of GDM.

Previous studies have consistently shown that older pregnant women are more likely to develop GDM, making advanced maternal age, usually defined as being 35 years or older, a well-documented, non-modifiable risk factor for this condition [6]. Other potential non-modifiable risk factors, although not always confirmed, include family history of T2DM, ethnicity, young age at menarche, polycystic ovary syndrome (PCOS), and other reproductive and endocrine disorders contributing to infertility and/or pregnancy complications [1]. Metabolic derangements caused by insulin resistance, such as GDM and T2DM, are closely linked to the aging process, as the whole-body intracellular responses to insulin tend to progressively decrease with age, often resulting in glucose intolerance [7,8]. However, there is a lot of variation in how the systemic, age-related dysfunction of insulin signaling can be affected by adiposity and hormonal products derived from visceral fat tissue. In regard to this, a recent translational study conducted by our group in a non-pregnant, gender-mixed population has demonstrated that the increase in body mass index (BMI), which reflects visceral fat accumulation and deregulation of adipose secretory functions, is actually the main driver of systemic insulin resistance in humans [9]. Despite a potential age-related deterioration [10], the molecular changes in insulin signaling caused by obesity are not permanent, but reversible with appropriate interventions [9]. Maternal preconception BMI has been identified as a potentially modifiable risk factor for GDM in numerous studies conducted in Europe, America, Australia, and Asia [11,12,13]. However, the effect of maternal preconception BMI on the development of GDM has often been seen in combination with the effects of gestational body weight gain and age, so it is still uncertain which of these factors contribute most to the onset of GDM [14]. Here, we aimed to investigate the relative impacts of modifiable and non-modifiable risk factors for GDM in a large retrospective population of pregnant women, with a special focus on maternal preconception BMI and age, both of which are key determinants of systemic insulin resistance. Understanding the factors that contribute to the current escalation of GDM rates in pregnant women could help to undertake prevention and intervention strategies, particularly in areas where this condition is more common, such as southern Italy.

## 2. Materials and Methods

### 2.1. Study Population 

In this monocentric, retrospective, population study, 3865 consecutive singleton pregnant women, attending the tertiary care Endocrinology Unit of “Pugliese Ciaccio” Hospital (Catanzaro, Italy) from January 2017 to March 2020, before the outbreak of the novel coronavirus (COVID-19) pandemic [15], for a 75 g OGTT screening test for GDM, were enrolled. Screening for GDM with a 75 g OGTT was performed at 16–18 and/or 24–28 weeks of gestation (wg), in accordance with the risk-factor-based guidelines endorsed by the Italian Ministry of Health [16]. In regard to this, it is worth noting that, in Italy, an anticipated screening for GDM is recommended for women with at least one of the following risk factors: (1) preconception obesity; (2) preconception and/or first trimester biochemical evidence of impaired fasting glucose (IFG, fasting plasma glucose levels of 100–125 mg/dL); or (3) previous GDM. Women who test negative for GDM at this early screening are recommended to be re-tested for GDM at 24–28 wg with another 75 g OGTT [16]. However, as widely documented by our group and others [17,18,19], in the real-world practice, only a minority of pregnant women with these risk factors undergoes an anticipated 75 g OGTT screening test for GDM at 16–18 wg. In most cases, the diagnosis of GDM follows a late, and almost universal, 75 g OGTT screening test at 24–28 wg, regardless of maternal risk factors [17,18,19]. During the early stages of the COVID-19 outbreak, screening strategies for GDM in Italy were temporarily changed in order to reduce the risk of coronavirus infection for pregnant women and limit unnecessary hospital accesses. In this emergency situation, the diagnosis of GDM was allowed to be based on fasting glucose values alone, to limit the number of 75 g OGTT screening tests, and thus, potential exposure times in hospitals [20]. However, in all women enrolled in this study, GDM was diagnosed when one or more of the venous plasma glucose values exceeded the IADPSG thresholds (fasting ≥ 92 mg/dL, 1 h after 75 g OGTT ≥ 180 mg/dL, and 2 h after 75 g OGTT ≥ 153 mg/dL) as per current Standard of Care [16]. The laboratory analytical details of the 75 g OGTT screening test for GDM in this diabetes care center have been reported elsewhere [19].

Maternal age, family history of T2DM (first- or second-degree relatives), previous GDM, gravidity, reproductive history of spontaneous abortion(s) (the type of abortion was not specified), type of conception (i.e., natural/planned/assisted), age of menarche, level of education, marital status, smoking status, treated and/or well-controlled comorbid endocrine conditions (i.e., thyroid disease), self-reported last menstrual period, and preconception body weight, together with core anthropometric measurements (i.e., height, body weight) obtained during a standard nursing work-up in the same morning of the 75 g OGTT, and venous plasma glucose results, were routinely recorded in an electronic patient diary (Smart Digital Clinic^®^, Meteda Srl) and retrospectively collected for the purpose of this study. The electronic patient diary automatically calculated the preconception BMI and gestational weight gain up to the time of the 75 g OGTT, taking into account the body weight (in kilograms) and height (in meters). BMI was therefore expressed with the formula body weight divided by height square (kg/m^2^), and gestational weight gain was calculated as the difference between body weight at the time of the 75 g OGTT and preconception body weight. Women who were under prophylactic heparin therapy at the time of the 75 g OGTT due to laboratory evidence of inherited (i.e., carriers of Factor V Leiden and Prothrombin G20210A variants, or with a congenital Protein S/C deficiency) or acquired (i.e., positive lupus anticoagulant and anticardiolipin antibodies) coagulative disorders, or with a positive history of venous thromboembolism in the preconception period, were considered as thrombophilic [16]. Exclusion criteria were multiple pregnancies, active chronic systemic diseases, or use of medications affecting glucose tolerance (i.e., metformin [21]). 

### 2.2. Statistical Analysis

Continuous traits were expressed as medians and interquartile ranges (IQRs), while categorical traits were expressed as numbers and percentages. The Mann–Whitney U test was used to determine the presence of significant differences in the distribution of continuous variables between women diagnosed with GDM and those without the condition. The Chi-square (χ^2^) test was used to compare proportions. To investigate the existence of a linear relationship between the continuous, near-normally shaped, potential predictors of GDM (i.e., maternal preconception BMI and age) and venous plasma glucose values at the time of the 75 g OGTT, Pearson correlation tests were performed, generating a heat map of correlation coefficients. For the univariate correlation analysis, missing data were handled with pairwise deletion. To further explore the influence of predictors on the likelihood of having a GDM diagnosis at the time of the 75 g OGTT, the available data regarding maternal preconception BMI and age were fitted into logistic regression models, with appropriate covariate adjustments. Adjusted odds ratios (OR) with 95% confidence intervals as relative effect estimates were calculated. A significance level of 0.05 was set for all analyses. Data were analyzed with JASP Graphical Statistical Software Version 0.17.1.0 (University of Amsterdam, Amsterdam, the Netherlands) based on R Stats packages.

## 3. Results

### 3.1. Characteristics of Pregnant Women and Risk Factors for Gestational Diabetes Mellitus (GDM)

Out of 3856 singleton pregnant women undergoing a 75 g OGTT during gestation, 885 (23.0%) were diagnosed with GDM according to IADPSG criteria. Table 1 shows the differences in clinical characteristics between women who were diagnosed with GDM and those who with normal glucose tolerance. Intergroup comparisons revealed that a significantly smaller proportion of women with GDM reported planning their pregnancy compared to women who were normal glucose tolerant (39.8% vs. 54.9%, *p* < 0.001). However, despite the amount of missing data for social and lifestyle variables, there were no significant differences in education, marital status, and smoking status between the two groups. In contrast, a family history of T2DM was more frequently reported in women with GDM (71.1% vs. 54.6%, *p* < 0.001). Women with GDM were, on average, 2 years older than normal glucose-tolerant women (median preconception maternal age: 34 vs. 32 years, *p* < 0.001). Additionally, a significantly greater proportion of women with GDM were of advanced age at conception (42.8% vs. 33.6%, *p* < 0.001), supporting the historical, well-documented, role of aging as a risk factor for GDM [22], whereas there were no significant differences in self-reported median age at menarche (12 years for both groups). In relation to obstetric characteristics, fewer women with GDM were found to be nulliparous (39.0% vs. 43.6%, *p* = 0.021), and had no reproductive history of spontaneous abortion(s) (75.2% vs. 80%, *p* = 0.003) in comparison to women with normal glucose tolerance. Furthermore, a significantly greater proportion of women with GDM had PCOS (5.0% vs. 0.7%, *p* < 0.001), and became pregnant through assisted reproductive technology (1.3% vs. 0.2%, *p* < 0.001). Additionally, women with GDM were, with respect to normal glucose-tolerant women, more than twice as likely to be affected by thyroid diseases (6.9% vs. 3.2%, *p* < 0.001), in some cases under treatment with substitutive hormone therapy, and by thrombophilic disorders (3.2% vs. 0.7%, *p* < 0.001). A significantly larger proportion of women with GDM had also been diagnosed with IFG before pregnancy (21.1% vs. 6.2%, *p* = 0.015), even though only a few women documented their pre-existing glucose tolerance status, and most data were missing. With regard to anthropometric variables, women with GDM tended to be slightly shorter (by an average of 1 cm) and have a greater body weight (median body weight: 65.0 vs. 60.0 kg, *p* < 0.001) than normal glucose tolerant women. Not surprisingly, women with GDM also had a preconception BMI that was ~3 points higher (median BMI: 25.0 vs. 22.7 kg/m^2^, *p* < 0.001), meaning that larger percentages of women with obesity (BMI ≥ 3.0 kg/m^2^) or overweight (BMI 25–29.9 kg/m^2^) were diagnosed with GDM. 

Previous GDM is the strongest risk factor for GDM recurrence [23]. In this study, a larger percentage of women with a history of GDM in a previous pregnancy was diagnosed again with this condition (17.4% vs. 3.4%, *p* < 0.001). However, only a minority of these at-risk women were diagnosed with GDM following an anticipated 75 g OGTT at 16–18 wg (12.8%), and in most cases, diagnosis of GDM occurred at 24–28 wg, because of poor adherence of pregnant women to early screening tests [17,18,19] (Table 1). Women diagnosed with GDM continued to have a significantly higher body weight than normal glucose-tolerant women at 24–28 wg (median body weight: 72.0 vs. 68.8 kg; median BMI: 27.7 vs. 25.6 kg/m^2^, *p* < 0.001), although without any differences in gestational body weight gain up to the scheduled 75 g OGTT date (median 7.0 kg for both groups). 

### 3.2. Effect of Maternal Preconception Body Mass Index and Age on GDM Risk

**Figure 1:**
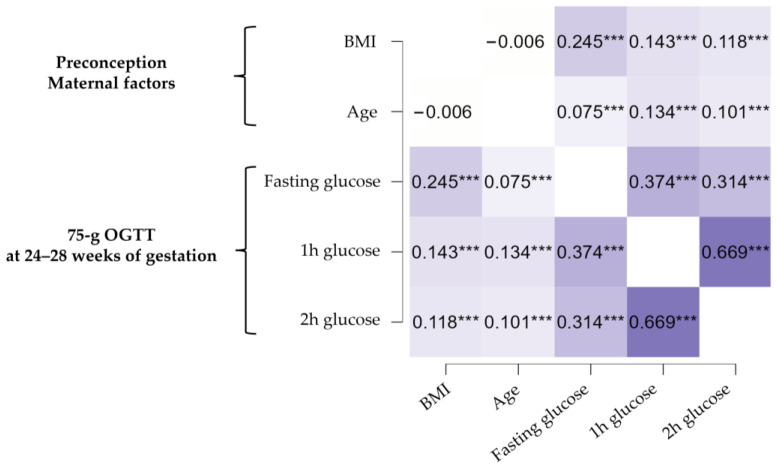
Heat map of Pearson’s correlation coefficients. *** indicates statistical significance with *p* < 0.001.

To elucidate the relationship between maternal preconception BMI, age, and the risk of developing GDM, univariate correlation analysis was initially employed. Figure 1 illustrates the lack of reciprocal association between maternal preconception BMI and age, as shown by the heat maps of Pearson’s correlation coefficients. However, both preconception maternal factors had a positive, but weak association with 1 h and 2 h post-load plasma glucose values resulting from the 75 g OGTT screening test performed at 24–28 wg (Pearson coefficients ranging from 0.101 to 0.143). Contrary to age, maternal preconception BMI had a moderate positive association with fasting glucose levels (Pearson coefficient: 0.245). This represents a relevant finding, because the majority (60%) of GDM diagnoses in this pregnant study population were based on abnormal fasting glucose levels, regardless of post-load glucose values (Table 1).

Then, after considering previous reports about the independent effects of both BMI and aging in determining systemic insulin resistance, and thus, glucose intolerance [6,7,8,9], logistic regression analysis was performed to determine more accurately which one of these factors could be a better predictor of GDM in pregnant women. In adjusted logistic regression models, controlling for gravidity, reproductive history of spontaneous abortion(s), GDM in a previous pregnancy, assisted conception, thyroid and thrombophilic diseases, PCOS, family history of T2DM, and gestational body weight gain, maternal preconception BMI was found to be a stronger determinant of GDM risk than maternal age, as evidenced by the higher absolute value of the standardized β coefficient (0.414 for preconception BMI vs. 0.246 for maternal age) (Table 2). 

Maternal preconception obesity almost tripled the risk of developing GDM (adjusted OR: 2.525, 95% CI 1.971–3.236, *p* < 0.001) (Table 3), but even preconception overweight was found to increase the risk of GDM more than advanced maternal age (adjusted OR for overweight: 1.63, 95% CI 1.320–2.019, *p* < 0.001; adjusted OR for advanced maternal age: 1.45, 95% CI 1.184–1.776, *p* < 0.001) (Table 4). 

Finally, the data in Table 5, which stratify the women into different risk groups based on maternal preconception BMI and age, suggest that prevention of overweight and obesity prior to pregnancy is the most appropriate strategy to reduce the number of cases of GDM in areas where it is currently highly prevalent, such as southern Italy. In fact, in this large, contemporary, study population, the prevalence of GDM increased dramatically as the maternal preconception BMI changed from normal weight to the overweight and obesity ranges, in all age groups (for women younger than 35 years, the prevalence of GDM increased from 16.4% to 23.0% to 38.5%; for women with an advanced maternal age, the prevalence of GDM increased from 20.4% to 37.2% to 51.4%). A crude pairwise comparison of sequentially decreasing risk categories showed that there was no statistically significant difference in the prevalence rate of GDM between overweight women with advanced maternal age and obese women younger than 35 years (*p* = 0.753), as well as between normal weight women with advanced maternal age and overweight women younger than 35 years (*p* = 0.255), although there was a nominal difference in disfavor of excess body weight in the latter case (Table 5). This finding corroborates the idea that being overweight before pregnancy is a relevant risk factor for GDM, even at a young maternal age. This excess risk can be equal to or greater than the risk associated with delayed childbearing, when other potential risk factors are more likely to be present.

## 4. Discussion

As more women enter pregnancy at an advanced maternal age and/or with excess body weight, the risk of developing GDM is steadily rising in most countries and regions of the world, especially in southern Italy [2], where one out of five women is currently diagnosed with this condition. According to national statistics, approximately 33% of adults in this area are affected by overweight or obesity, including women [24]. Women tend to plan their pregnancies at 35 years or older, which may be due to a desire to complete their education and professional development, as well as to a delayed formation of stable households [19].

Clinical trials support the notion that aging, in general, is associated with systemic insulin resistance and an increased risk of developing T2DM [7,8], or, in case of women in their reproductive years, GDM [6]. Although there are some exceptions to this rule [25], with age, people tend to have less lean body mass (particularly skeletal muscle tissue) and more visceral fat mass. Since skeletal muscle is a main site of insulin-stimulated glucose uptake, decreased muscle mass can lead to decreased whole-body glucose disposal and, as a result, glucose intolerance [8,25]. 

In late gestation, it is known that there is a physiological decrease in skeletal muscle insulin sensitivity, which determines a reduction in insulin-stimulated whole-body glucose disposal by 50% [26]. These metabolic changes occur independently of a diagnosis of GDM. They are, from an evolutionary point of view, designed to limit maternal glucose utilization and thereby shunt an adequate amount of supply to the growing fetus, which requires glucose as its major energy source [2]. In women with normal glucose tolerance, the changes in insulin sensitivity are, however, balanced by an adequate increase in insulin production from maternal pancreatic β cells, whereas in women with GDM, insulin secretion is relatively insufficient to compensate for the various degrees of systemic insulin resistance [1]. In regard to this, it has been studied that, in glucose-intolerant, non-pregnant women, the ability to secrete insulin decreases by 0.7% per year, as a consequence of accelerated pancreatic β cell dysfunction [27]. Additionally, the aging process negatively affects the capacity of pancreatic β cells to proliferate during gestation [28], potentially facilitating the development of GDM in pregnant women.

Although some historical lines of evidence suggest that age is correlated with insulin sensitivity [25], it does not appear to be the primary factor affecting whole-body responses to insulin. Age-related changes in anthropometric parameters related to skeletal muscle and visceral fat masses are the true responsible for the increase in systemic insulin resistance [8,25,29]. In particular, the expansion of visceral fat is a major contributing factor, with several reports showing linear correlations between visceral adiposity and insulin resistance in humans [8,25,29]. The precise mechanisms by which an enlarged visceral fat mass should cause systemic insulin resistance are not yet fully understood. The most recent experimental data from our group on this topic are in support of the pathogenic role of hypoxia [9]. Visceral adipocytes from obese individuals have a reduced content of oxygen. When exposed to hypoxia, adipocytes have a reduced capacity to uptake glucose under insulin stimulation, and to contribute, with skeletal myocytes, to whole-body glucose disposal [9]. Adipocytes from obese individuals have also a dysfunctional secretory activity, which gets progressively worse as the BMI increases, reflecting greater expansion of visceral fat tissue and more severe oxygen deficits [9]. In non-pregnant situations, the circulating levels of adipokines and cytokines, notoriously linked to insulin resistance and energy balance regulation, such as tumor necrosis factor α, several proinflammatory interleukins, and chemoattractant proteins (i.e., MCP-1), plasminogen-activator inhibitor-1, retinol binding protein 4, resistin, and leptin, showed a positive relationship with BMI [9]. In recent years, studies have begun to investigate whether changes in levels of circulating adipokines and cytokines also play a role in the development of GDM [30]. However, their relationships with maternal preconception BMI and age are, presently, undetermined, as both placenta and visceral fat contribute to the circulating levels of adipokines and cytokines in pregnant women [30].

The current hypothesis that in women, the likelihood of developing systemic insulin resistance during pregnancy may be predetermined by her genetic heritage is supported in this study by the fact that non-modifiable factors, such as PCOS and a family history of T2DM, two conditions that contribute to systemic insulin resistance in the presence of a negative environment (i.e., unhealthy dietary habits and lifestyles) and of genetic overlaps [30], were more common in women with GDM than in those with normal glucose tolerance. Furthermore, in this study, women with GDM were more frequently affected by thyroid diseases (namely caused by thyroid autoimmunity and/or goiter), suggesting that this non-modifiable factor could be linked with GDM development. A previous clinical investigation, which found that thyroid dysfunction is associated with GDM, even after adjusting for advanced maternal age and obesity [31], is in line with the present findings, and hints that a causal association between thyroid diseases and insulin resistance in pregnancy might exist.

Women who have had abortions in previous pregnancies [32,33], who are affected by thrombophilic disorders [34], or who have conceived using assisted reproductive technologies [32,35] are more susceptible to developing pregnancy diseases attributable to the placenta. GDM is a major cause of placental dysfunction, but, at the same time, it is caused by an abnormal release of hormones, metabolites, and cytokines from this organ [1,2,30]. Placenta-related factors may contribute to the reduction of insulin sensitivity and cause inflammation in pregnant women, potentially exacerbating the effect of excess BMI and advanced maternal age [30].

The findings of this study indicate that maternal preconception BMI and age are both independently associated with GDM, even after adjusting for all the potential confounders, which include many non-modifiable risk factors (family history of T2DM, PCOS, previous GDM, gravidity, assisted reproduction, history of abortions, thyroid and thrombophilic diseases) and a potentially modifiable one (gestational weight gain). Maternal age and overweight/obesity have been demonstrated to be independent determinants of the risk of GDM in many studies [11,12,13]. In contrast, there is conflicting evidence regarding the independent effect of gestational weight gain in the development of GDM. A recent study on Malay women found no association between first and second trimester gestational weight gain and the risk of GDM [14], which contradicts other previous studies that have found such an association, especially among obese women and women aged ≥ 35 years [36,37]. Here, there was no significant difference in gestational weight gain up to the time of 75 g OGTT between women with GDM and normal glucose-tolerant women. In addition, the total median amount of gestational weight gain was within the expected range for a physiological pregnancy [38]. These findings suggest that interventions to prevent GDM (i.e., lifestyle-based) may be most effective if implemented during the preconception period, rather than during the first or second trimester, in populations where the condition is highly prevalent due to advanced maternal age, genetic factors, and high rates of overweight or obesity, as in Malay and southern Italian women.

For interventions that aim to improve public health, it is necessary to have knowledge of both population- and individual-level risks. By searching the modifiable and non-modifiable factors that may predispose women to develop GDM, we can target actions that may reduce the risk of developing this condition and its associated maternal and fetal complications [1]. Presently, there is growing consensus that women who are at higher risk for developing GDM should have a 75 g OGTT earlier in pregnancy and should receive more intensive interventions in order to effectively prevent GDM [17,39,40]. This is especially relevant for obese women, who we have previously reported to experience a more severe degree of gestational insulin resistance and hyperglycemia than non-obese women. The result is an excessive amount of glucose being transferred to the fetus, which can cause irreversible acceleration of its growth trajectory and complications, even before GDM is diagnosed [17]. However, no known universal system or algorithm exists to accurately predict the development of GDM or to define the optimal timing for preventive intervention and diagnostic testing (i.e., early vs. late gestation) [39,40]. The research in this area is constantly evolving and may eventually provide more reliable results than the current risk-factor-based guidelines, such as the one endorsed by the Italian Ministry of Health [41].

In the final part of the study, pregnant women were categorized into six risk groups based on their maternal preconception BMI and age. The risk group with the highest prevalence of GDM consisted of obese women aged 35 years or older. The prevalence of GDM was similar for obese women aged less than 35 years and for overweight women 35 years or older, while the prevalence of GDM was somewhat greater for younger overweight women than for normal weight women of advanced maternal age. Overall, these findings support the idea that excess BMI prior to conception can have a negative effect on insulin resistance, predisposing to GDM, and this effect is comparable to, or even greater than, the effect of advanced maternal age.

This is the first study to investigate the relative effects of preconception BMI and maternal age on the risk of GDM in southern Italy, taking into consideration the contemporary characteristics of women in this area and their increasing trend to postpone childbearing. Although there has been some progress in raising awareness of the obstetric and perinatal risks associated with pregnancy at advanced maternal age [42], there is still much work to be done to educate women in their reproductive years about the risks related to a high preconception BMI. Many women are unaware of the reproductive and metabolic problems that might occur being overweight or obese, often misjudging their own body size or prioritizing appearance over health [43,44]. However, as indicated in this study, the risk of developing GDM is more impacted by preconception BMI rather than age. This excess risk can be potentially reduced through lifestyle interventions that lead to weight loss.

In this work, there are some limitations. First, it is a retrospective study, with missing data from social and lifestyle variables, and second, the information about preconception body weight and reproductive history of pregnant women is self-reported. Another limitation is that the results may not be necessarily generalizable to populations in which GDM is less common or diagnosed using different criteria than those of the IADPSG. Additionally, we did not assess the relative impact of preconception BMI and maternal age on the risk of adverse pregnancy outcomes and perinatal morbidity, and this limits the possibility of making recommendations for changes to the current screening strategy for GDM adopted in southern Italy, as in the rest of the country. Presently, it is not yet possible to recommend an anticipated 75 g OGTT in overweight women older than 35 years [19], although they possess the same risk of developing GDM as younger women with preconception obesity. However, its monocentric design, with all pregnant women being enrolled and performing 75 g OGTT at the same diabetes care center, is a strength that helps to reduce the risk of biases associated with inter-laboratory analytical variations [19], as well as the misclassification of risk factors related to inter-operator variability in the recording of relevant medical information during routine diagnostic work-ups [45].

## 5. Conclusions

This study provides additional evidence that maternal preconception BMI, which is indicative of visceral adiposity and systemic insulin resistance, has a critical role in determining GDM. Unlike maternal age, preconception BMI is a modifiable risk factor, making it an appropriate target for implementing dedicated public health policies. Being the most common disorder among pregnant women, taking action to address this issue is essential in order to counteract the current rise in GDM rates in some geographic areas, including southern Italy, in which GDM prevalence is particularly high.

## Figures and Tables

**Table 1:** Comparison of the characteristics of pregnant women with gestational diabetes mellitus (GDM) to those of normal glucose-tolerant women (NGT).

Characteristics	NGT (*n* = 2980)	GDM (*n* = 885)	
	Median (IQR) or N (%)	Median (IQR) or N (%)	*p* Value
Secondary or tertiary level education, N	1871 (85.8% ^§^)	496 (83.2% ^§^)	0.099
Married, N	1709 (76.1% ^§^)	446 (78.5% ^§^)	0.222
Height, m	1.62 (1.59–1.67)	1.62 (1.58–1.65)	**0.001**
Preconception body weight, kg	60.0 (54.0–69.0)	65.0 (57.0–77.8)	**<0.001**
Preconception BMI, kg/m^2^	22.7 (20.6–25.7)	25.0 (21.9–29.4)	**<0.001**
Preconception obesity, N	241 (8.8% ^§^)	181 (21.9% ^§^)	**<0.001**
Preconception overweight, N	571 (20.7% ^§^)	224 (27.1% ^§^)	**<0.001**
Preconception IFG, N	8 (6.1% ^§^)	12 (21.1 ^§^)	**0.015**
PCOS, N	20 (0.7%)	44 (5.0%)	**<0.001**
Age at menarche, yr	12 (11–13)	12 (11–13)	0.717
Maternal age, yr	32 (29–36)	34 (30–37)	**<0.001**
Advanced maternal age (≥35 yr), N	1000 (33.6%)	379 (42.8%)	**<0.001**
Thyroid disease, N	94 (3.2% ^§^)	61 (6.9% ^§^)	**<0.001**
Thrombophilia, N	21 (0.7% ^§^)	28 (3.2% ^§^)	**<0.001**
Nulliparous, N	1210 (43.6% ^§^)	318 (39.0% ^§^)	**0.021**
Negative reproductive history of abortion(s), N	2205 (80.0% ^§^)	613 (75.2% ^§^)	**0.003**
Planned pregnancy, N	1445 (54.9% ^§^)	310 (39.8% ^§^)	**<0.001**
Assisted reproduction, N	6 (0.2% ^§^)	12 (1.3% ^§^)	**<0.001**
Previous GDM, N	102 (3.4% ^§^)	153 (17.4% ^§^)	**<0.001**
Non-smoker, N	1904 (81.6% ^§^)	499 (82.3% ^§^)	0.185
Family history of T2DM, N	1574 (54.6% ^§^)	601 (71.1% ^§^)	**<0.001**
Diagnosis of GDM at early screening, N	_	113 (12.8%)	_
Gestational age at 75 g OGTT, wg	26.0 (26.0–27.0)	26.0 (25.0–27.0)	0.541
Fasting glucose *, mg/dL	80.0 (76.0–84.0)	92.0 (85.0–96.0)	**<0.001**
1 h glucose *, mg/dL	126.0 (107.0–145.0)	176.0 (149.0–192.0)	**<0.001**
2 h glucose *, mg/dL	102.0 (89.0–115.0)	134.0 (115.0–157.0)	**<0.001**
Fasting glucose ≥ 92 mg/dL, N	_	531 (60%)	_
1 h glucose ≥ 180 mg/dL, N	_	401 (45.3%)	_
2 h glucose ≥ 153 mg/dL, N	_	276 (31.2%)	_
Body weight at 75 g OGTT *, kg	68.0 (61.0–76.0)	72.0 (64.0–83.0)	**<0.001**
BMI at 75 g OGTT *, kg/m^2^	25.6 (23.4–28.5)	27.7 (24.7–31.6)	**<0.001**
Gestational weight gain at 75 g OGTT *, kg	7.0 (5.0–9.0)	7.0 (5.0–10.0)	0.992

Data are expressed as medians and interquartile ranges (IQRs) or as numbers and percentages. “^§^” signs indicate valid percentages computed on the total number of non-missing values for the specific variable. *p*-values were calculated using the Mann–Whitney U test or the χ^2^ test, as appropriate. Bold values denote statistical significance at *p* < 0.05. “*” signs indicate that the high-risk women diagnosed with GDM at early screening (i.e., a 75 g OGTT performed at 16–18 wg) as per Italian Ministry of Health guidelines [16] were excluded from the analysis. Women who stopped smoking during pregnancy but were smokers in the preconception period were considered as “smokers”. IFG, impaired fasting glucose; PCOS, polycystic ovary syndrome; T2DM, type 2 diabetes mellitus.

**Table 2:** Logistic regression analysis showing the independent effects of maternal preconception body mass index (BMI) and age in predicting a diagnosis of GDM. Data are expressed as crude and adjusted odds ratios (OR).

	Standardized β	OR	95% CI	*p* Value
Preconception BMI	0.481	1.104	(1.087–1.122)	**<0.001**
Preconception BMI *	0.414	1.089	(1.069–1.109)	**<0.001**
Maternal age	0.279	1.055	(1.038–1.072)	**<0.001**
Maternal age *	0.246	1.048	(1.029–1.068)	**<0.001**

* OR was adjusted by forcing gravidity, reproductive history of spontaneous abortion(s), previous GDM, assisted reproduction, thyroid and thrombophilic diseases, PCOS, family history of T2DM, and gestational bodyweight gain as covariates in the logistic regression model, in consideration of the results of univariate analyses (Table 1) and previous literature reports [14]. Multicollinearity in logistic regression analysis was tested by evaluation of Variance Inflation Factors (VIF). All VIF measures were <2.5. Bold values denote statistical significance at *p* < 0.05.

**Table 3:** Logistic regression analysis showing the independent effects of preconception obesity and advanced maternal age in predicting a diagnosis of GDM. Data are expressed as adjusted OR.

	Standardized β	OR	95% CI	*p* Value
Preconception obesity	0.299	2.525	(1.971–3.236)	**<0.001**
Maternal age ≥ 35 yr	0.181	1.461	(1.213–1.759)	**<0.001**

Gravidity, reproductive history of spontaneous abortion(s), previous GDM, assisted reproduction, thyroid and thrombophilic diseases, PCOS, family history of T2DM, and gestational bodyweight gain were used as covariates in the logistic regression model. Bold values denote statistical significance at *p* < 0.05.

**Table 4:** Logistic regression analysis showing the independent effects of preconception overweight and advanced maternal age in predicting a diagnosis of GDM. Data are expressed as adjusted OR.

	Standardized β	OR	95% CI	*p* Value
Preconception overweight	0.108	1.633	(1.320–2.019)	**<0.001**
Maternal age ≥ 35 yr	0.103	1.450	(1.184–1.776)	**<0.001**

Gravidity, reproductive history of spontaneous abortion(s), previous GDM, assisted reproduction, thyroid and thrombophilic diseases, PCOS, family history of T2DM, and gestational bodyweight gain were used as covariates in the logistic regression model. Bold values denote statistical significance at *p* < 0.05. Women with preconception obesity (*n* = 422) were excluded.

**Table 5:** Prevalence rates of GDM in risk groups of pregnant women stratified by preconception BMI and maternal age.

	Preconception Obesity				Preconception Overweight				Preconception Normal Weight				A vs. B	B vs. C	C vs. D	D vs. E	E vs. F
	(A) Maternal Age ≥35 yr		(B) Maternal Age <35 yr		(C) Maternal Age ≥35 yr		(D) Maternal Age <35 yr		(E) Maternal Age ≥35 yr		(F) Maternal Age <35 yr						
	N	%	N	%	N	%	N	%	N	%	N	%	*p* Value				
NGT	67	48.6%	174	61.5%	182	62.8%	389	77.0%	681	79.6%	1258	83.6%	*	ns	***	ns	*
GDM	71	51.4%	109	38.5%	108	37.2%	116	23.0%	174	20.4%	247	16.4%					
Total	138		283		290		505		855		1505						

Preconception obesity was defined by a BMI ≥ 30 kg/m^2^, preconception overweight by a BMI 25–29.9 kg/m^2^, preconception normal weight by a BMI < 25 kg/m^2^. * indicates statistical significance with *p* < 0.05; *** indicates statistical significance with *p* < 0.001; ns: non-significant; *p* values were calculated using the χ^2^ test. Women with missing values for one or both preconception variables (*n* = 280) were excluded.

## Data Availability

Data supporting the reported results are available from the corresponding author upon reasonable request.

## References

[1] Chiefari E., Arcidiacono B., Foti D., Brunetti A. (2017). Gestational diabetes mellitus: An updated overview. J. Endocrinol. Investig..

[2] Mirabelli M., Chiefari E., Tocci V., Greco E., Foti D., Brunetti A. (2021). Gestational diabetes: Implications for fetal growth, intervention timing, and treatment options. Curr. Opin. Pharmacol..

[3] Li G., Wei T., Ni W., Zhang A., Zhang J., Xing Y., Xing Q. (2020). Incidence and Risk Factors of Gestational Diabetes Mellitus: A Prospective Cohort Study in Qingdao, China. Front. Endocrinol..

[4] International Diabetes Federation (2021). IDF Atlas.

[5] Capula C., Chiefari E., Vero A., Iiritano S., Arcidiacono B., Puccio L., Pullano V., Foti D., Brunetti A., Vero R. (2013). Predictors of postpartum glucose tolerance testing in italian women with gestational diabetes mellitus. ISRN Endocrinol..

[6] Li Y., Ren X., He L., Li J., Zhang S., Chen W. (2020). Maternal age and the risk of gestational diabetes mellitus: A systematic review and meta-analysis of over 120 million participants. Diabetes Res. Clin. Pract..

[7] Chia C.W., Egan J.M., Ferrucci L. (2018). Age-Related Changes in Glucose Metabolism, Hyperglycemia, and Cardiovascular Risk. Circ. Res..

[8] Ferrannini E., Vichi S., Beck-Nielsen H., Laakso M., Paolisso G., Smith U. (1996). Insulin action and age. European Group for the Study of Insulin Resistance (EGIR). Diabetes.

[9] Arcidiacono B., Chiefari E., Foryst-Ludwig A., Currò G., Navarra G., Brunetti F.S., Mirabelli M., Corigliano D.M., Kintscher U., Britti D. (2020). Obesity-related hypoxia via miR-128 decreases insulin-receptor expression in human and mouse adipose tissue promoting systemic insulin resistance. EBioMedicine.

[10] Guo X., Asthana P., Gurung S., Zhang S., Wong S.K.K., Fallah S., Chow C.F.W., Che S., Zhai L., Wang Z. (2022). Regulation of age-associated insulin resistance by MT1-MMP-mediated cleavage of insulin receptor. Nat. Commun..

[11] Kim S.Y., England L., Wilson H.G., Bish C., Satten G.A., Dietz P. (2010). Percentage of gestational diabetes mellitus attributable to overweight and obesity. Am. J. Public Health.

[12] Chu S.Y., Callaghan W.M., Kim S.Y., Schmid C.H., Lau J., England L.J., Dietz P.M. (2007). Maternal obesity and risk of gestational diabetes mellitus. Diabetes Care.

[13] Mnatzaganian G., Woodward M., McIntyre H.D., Ma L., Yuen N., He F., Nightingale H., Xu T., Huxley R.R. (2022). Trends in percentages of gestational diabetes mellitus attributable to overweight, obesity, and morbid obesity in regional Victoria: An eight-year population-based panel study. BMC Pregnancy Childbirth.

[14] Yong H.Y., Shariff Z.M., Yusof B.N.M., Rejali Z., Tee Y.Y.S., Bindels J., van der Beek E.M. (2020). Independent and combined effects of age, body mass index and gestational weight gain on the risk of gestational diabetes mellitus. Sci. Rep..

[15] Mirabelli M., Chiefari E., Puccio L., Foti D.P., Brunetti A. (2020). Potential Benefits and Harms of Novel Antidiabetic Drugs During COVID-19 Crisis. Int. J. Environ. Res. Public Health.

[16] Linee Guida per la Gravidanza Fisiologica. Sistema Nazionale per le Linee Guida dell’Istituto Superiore di Sanità.

[17] Chiefari E., Quaresima P., Visconti F., Mirabelli M., Brunetti A. (2020). Gestational diabetes and fetal overgrowth: Time to rethink screening guidelines. Lancet Diabetes Endocrinol..

[18] Bianchi C., de Gennaro G., Romano M., Battini L., Aragona M., Corfini M., Del Prato S., Bertolotto A. (2017). Italian national guidelines for the screening of gestational diabetes: Time for a critical appraisal?. Nutr. Metab. Cardiovasc. Dis..

[19] Quaresima P., Visconti F., Chiefari E., Mirabelli M., Borelli M., Caroleo P., Foti D., Puccio L., Venturella R., Di Carlo C. (2020). Appropriate Timing of Gestational Diabetes Mellitus Diagnosis in Medium- and Low-Risk Women: Effectiveness of the Italian NHS Recommendations in Preventing Fetal Macrosomia. J. Diabetes Res..

[20] Torlone E., Festa C., Formoso G., Scavini M., Sculli M., Succurro E., Sciacca L., Lapolla A. (2020). Raccomandazioni per la diagnosi del diabete gestazionale durante la pandemia COVID-19. JAMD.

[21] Salatino A., Mirabelli M., Chiefari E., Greco M., Di Vito A., Bonapace G., Brunetti F.S., Crocerossa F., Epstein A.L., Foti D.P. (2022). The anticancer effects of Metformin in the male germ tumor SEM-1 cell line are mediated by HMGA1. Front. Endocrinol..

[22] Lao T.T., Ho L.F., Chan B.C., Leung W.C. (2006). Maternal age and prevalence of gestational diabetes mellitus. Diabetes Care.

[23] Coustan D.R. (2016). Recurrent GDM and the development of type 2 diabetes have similar risk factors. Endocrine.

[24] Mirabelli M., Chiefari E., Caroleo P., Vero R., Brunetti F.S., Corigliano D.M., Arcidiacono B., Foti D.P., Puccio L., Brunetti A. (2019). Long-Term Effectiveness and Safety of SGLT-2 Inhibitors in an Italian Cohort of Patients with Type 2 Diabetes Mellitus. J. Diabetes Res..

[25] Barbieri M., Rizzo M.R., Manzella D., Paolisso G. (2001). Age-related insulin resistance: Is it an obligatory finding? The lesson from healthy centenarians. Diabetes Metab. Res. Rev..

[26] Barbour L.A., McCurdy C.E., Hernandez T.L., Kirwan J.P., Catalano P.M., Friedman J.E. (2007). Cellular mechanisms for insulin resistance in normal pregnancy and gestational diabetes [published correction appears in Diabetes Care. 2007;30:3154]. Diabetes Care.

[27] Szoke E., Shrayyef M.Z., Messing S., Woerle H.J., van Haeften T.W., Meyer C., Mitrakou A., Pimenta W., Gerich J.E. (2008). Effect of aging on glucose homeostasis: Accelerated deterioration of beta-cell function in individuals with impaired glucose tolerance. Diabetes Care.

[28] Rieck S., Kaestner K.H. (2010). Expansion of beta-cell mass in response to pregnancy. Trends Endocrinol. Metab..

[29] Gautier J.-F., Mourier A., De Kerviler E., Tarentola A., Bigard A.X., Villette J.-M., Guezennec C.Y., Cathelineau G. (1998). Evaluation of abdominal fat distribution in noninsulin-dependent diabetes mellitus: Relationship to insulin resistance. J. Clin. Endocrinol. Metab..

[30] Kampmann U., Knorr S., Fuglsang J., Ovesen P. (2019). Determinants of Maternal Insulin Resistance during Pregnancy: An Updated Overview. J. Diabetes Res..

[31] Sitoris G., Veltri F., Ichiche M., Kleynen P., Praet J.-P., Rozenberg S., Poppe K.G. (2022). Association between thyroid autoimmunity and gestational diabetes mellitus in euthyroid women. Eur. Thyroid. J..

[32] Sun H., Su X., Liu Y., Li G., Liu X., Du Q. (2022). Association between Abortion History and Perinatal and Neonatal Outcomes of Singleton Pregnancies after Assisted Reproductive Technology. J. Clin. Med..

[33] Wang H., Guo X., Song Q., Su W., Meng M., Sun C., Li N., Liang Q., Qu G., Liang M. (2023). Association between the history of abortion and gestational diabetes mellitus: A meta-analysis. Endocrine.

[34] Hoffmann E., Hedlund E., Perin T., Lyndrup J. (2012). Is thrombophilia a risk factor for placenta-mediated pregnancy complications?. Arch. Gynecol. Obstet..

[35] Manna C., Lacconi V., Rizzo G., De Lorenzo A., Massimiani M. (2022). Placental Dysfunction in Assisted Reproductive Pregnancies: Perinatal, Neonatal and Adult Life Outcomes. Int. J. Mol. Sci..

[36] Dong B., Yu H., Wei Q., Zhi M., Wu C., Zhu X., Li L. (2017). The effect of pre-pregnancy body mass index and excessive gestational weight gain on the risk of gestational diabetes in advanced maternal age. Oncotarget.

[37] Hedderson M.M., Gunderson E.P., Ferrara A. (2010). Gestational weight gain and risk of gestational diabetes mellitus. Obstet. Gynecol..

[38] Rasmussen K.M., Yaktine A.L., Institute of Medicine (US) and National Research Council (US) Committee to Reexamine IOM Pregnancy Weight Guidelines (2009). Weight Gain During Pregnancy: Reexamining the Guidelines.

[39] Sparks J.R., Ghildayal N., Hivert M.F., Redman L.M. (2022). Lifestyle interventions in pregnancy targeting GDM prevention: Looking ahead to precision medicine. Diabetologia.

[40] Mirabelli M., Chiefari E., Foti D., Brunetti A. (2023). Preventing gestational diabetes mellitus with physical activity: Time to move towards precision medicine. L’Endocrinologo.

[41] Capula C., Chiefari E., Borelli M., Oliverio R., Vero A., Foti D., Puccio L., Vero R., Brunetti A. (2016). A new predictive tool for the early risk assessment of gestational diabetes mellitus. Prim. Care Diabetes.

[42] Temmesen C.G., Faber Frandsen T., Svarre-Nielsen H., Petersen K.B., Clemensen J., Andersen H.L.M. (2023). Women’s reflections on timing of motherhood: A meta-synthesis of qualitative evidence. Reprod. Health.

[43] Zelenytė V., Valius L., Domeikienė A., Gudaitytė R., Endzinas Ž., Šumskas L., Maleckas A. (2021). Body size perception, knowledge about obesity and factors associated with lifestyle change among patients, health care professionals and public health experts. BMC Fam. Pract..

[44] Sand A.S., Emaus N., Lian O. (2015). Overweight and obesity in young adult women: A matter of health or appearance? The Tromsø study: Fit futures. Int. J. Qual. Stud. Health Well-Being.

[45] Tajgardoon M., Cooper G.F., King A.J., Clermont G., Hochheiser H., Hauskrecht M., Sittig D.F., Visweswaran S. (2020). Modeling physician variability to prioritize relevant medical record information. JAMIA Open.

